# Aviator: a web service for monitoring the availability of web services

**DOI:** 10.1093/nar/gkab396

**Published:** 2021-05-26

**Authors:** Tobias Fehlmann, Fabian Kern, Pascal Hirsch, Robin Steinhaus, Dominik Seelow, Andreas Keller

**Affiliations:** Chair for Clinical Bioinformatics, Saarland University, 66123 Saarbrücken, Germany; Chair for Clinical Bioinformatics, Saarland University, 66123 Saarbrücken, Germany; Chair for Clinical Bioinformatics, Saarland University, 66123 Saarbrücken, Germany; Berlin Institute of Health at Charité - Universitätsmedizin Berlin, 10117 Berlin, Germany; Institute of Medical Genetics and Human Genetics, Charité - Universitätsmedizin Berlin, 13353 Berlin, Germany; Berlin Institute of Health at Charité - Universitätsmedizin Berlin, 10117 Berlin, Germany; Institute of Medical Genetics and Human Genetics, Charité - Universitätsmedizin Berlin, 13353 Berlin, Germany; Chair for Clinical Bioinformatics, Saarland University, 66123 Saarbrücken, Germany; Center for Bioinformatics, Saarland Informatics Campus, Saarland University, 66123 Saarbrücken, Germany; Department of Neurology and Neurological Sciences, Stanford University School of Medicine, Stanford, CA, USA

## Abstract

With Aviator, we present a web service and repository that facilitates surveillance of online tools. Aviator consists of a user-friendly website and two modules, a literature-mining based general and a manually curated module. The general module currently checks 9417 websites twice a day with respect to their availability and stores many features (frontend and backend response time, required RAM and size of the web page, security certificates, analytic tools and trackers embedded in the webpage and others) in a data warehouse. Aviator is also equipped with an analysis functionality, for example authors can check and evaluate the availability of their own tools or those of their peers. Likewise, users can check the availability of a certain tool they intend to use in research or teaching to avoid including unstable tools. The curated section of Aviator offers additional services. We provide API snippets for common programming languages (Perl, PHP, Python, JavaScript) as well as an OpenAPI documentation for embedding in the backend of own web services for an automatic test of their function. We query the respective APIs twice a day and send automated notifications in case of an unexpected result. Naturally, the same analysis functionality as for the literature-based module is available for the curated section. Aviator can freely be used at https://www.ccb.uni-saarland.de/aviator.

## Graphical Abstract

**Graphical Abstract:**
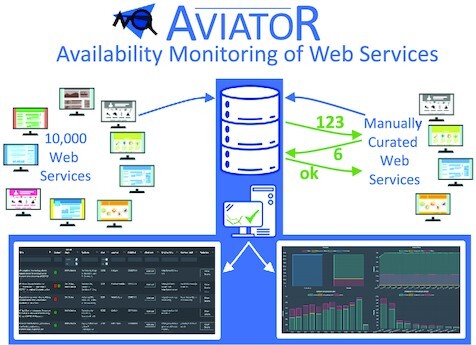
Aviator tracks the availability of other web services and provides interactive visualizations for daily updated statistics.

## INTRODUCTION

Undisputedly, web servers and web services as well as databases are of tremendous value for researchers and research projects worldwide. Software packages and frameworks such as Shiny from R, the Python-based Django framework, the likewise Python-based Flask package, Ruby on Rails and many alternative solutions facilitate the easy development and deployment of professional web-based tools. An issue that becomes increasingly relevant is the availability and sustainability of respective solutions. Certainly, the very successful tools and resources such as the web service to the basic local alignment search tool BLAST, originally published by Altschul in 1990 (1), Gapped BLAST and PSI-blast (2,3), STRING (4–6), the collection of tools at the European Bioinformatics Institute (EMBL-EBI) (7) or the Gene Ontology Database (8) are regularly updated and maintained. Other resources, such as the Kyoto Encyclopedia of genes and genomes KEGG (9–11) are also available under commercial licenses. In the light of a steadily increasing complexity in research and increasing data set sizes, common standards and scientific guidelines are mandatory for data- and code sharing practices. ELIXIR, one of the largest multi-national endeavors to integrate and coordinate computing facilities, web services, and databases across >220 research organizations has become a lighthouse in this respect (12). As part of ELIXIR, the FAIRsharing service provides community-based and reviewed standards/policies for sharing and maintaining databases (13). The aforementioned availability of successful tools in the community is self-evident. Vice versa, the availability of more narrow and specialized web services with a limited user community partially remains a challenge. A possible lack of persistence, usability, and functionality has thus been investigated by Veretnik and colleagues already in 2008 (14) on manuscripts published in the Nucleic Acids Research Web Server Issue from the previous four years. From 2003 onwards, this special issue became one of the most relevant source for web servers (15–19). In 2011, Schultheiss and co-workers presented an extended analysis on 927 tools in the special issue (20).

Nevertheless, also other journals publish tools and databases, calling for a broader analysis of the availability and functionality. We thus performed a large analysis and collected 2727 articles describing 2396 unique tools published by PubMed indexed journals from 2010 onwards (21). We checked the reachability of these tools and tested their availability over time. From our experience in this project, we implemented the Aviator web service. Aviator aims to be the most comprehensive and regularly (automatically) updated resource for recording availability of web servers and web services over time. Other resources maintaining collections of web-based tools are e.g. https://www.biostars.org or https://bioinformaticssoftwareandtools.co.in/, but they do not focus on the availability of these tools. In this direction, the EMBRACE Registry has been developed (22) but is unfortunately not available anymore (http://www.embraceregistry.net). To overcome challenges as described by Goble (23) and others, Aviator consists of two modules, an automated and literature mining-based section as well as a curated part. Using Aviator, authors can check the performance of their own and other tools as well as identify potential availability gaps. Likewise, it can facilitate or support interesting scientometric studies (24,25).

## MATERIALS AND METHODS

### Data collection

Websites and publications were retrieved from bio.tools (26) and PubMed. We downloaded all tools listed as ‘Web service’, ‘Web API’, ‘Web application’, ‘Bioinformatics portal’ and ‘Database portal’ from the bio.tools website (latest updated on 2021-02-09) and kept all entries that could be linked to a publication (either with a PMID or with a DOI that could be converted to a PMID). In a second step, we used the Biopython Entrez API (27) to download all publications from 2010 onwards from PubMed, in which the following terms were present in the title or abstract: webserver, web server, web-server, web service, web-service, web portal, web-portal, online tool, website, web based, web-based, web interface, web-interface, web tool, web-tool, network service, network-service, server. In addition, we included all publications from the Nucleic Acids Web Server Issue from 2010 onwards. Subsequently, all publications were filtered for those with at least one URL in their abstract. An additional semi-automatic filtering was then performed on the remaining publications to filter out false positives stemming from e.g., clinical trial registrations or pointing only to source code repositories and not at an actual web repository (examples include GitHub, Bitbucket, Google Code and GitLab). For the remaining publications, the corresponding author email address was determined via the Web of Science. All collected URLs were then normalized to collapse all addresses differing only by their protocol or by a trailing slash, privileging URLs accessible over https, as well as URLs without trailing slash.

### Website availability testing

To test the availability of a web server we set up a crawler that tries to reach the webpage twice per day. We implemented a python script running Selenium (3.141.0) with Google Chrome and ChromeDriver (85.0.4183.83) and displayed via pyvirtualdisplay (1.3.2) inside a Docker container. When querying a webpage our implementation waits up to 30 s and additional 30 s in case status code 202 is returned, which is frequently the case for launching R Shiny servers. Performance metrics such as the backend and frontend response time or the memory usage are extracted via the JavaScript console. Other information such as the number of requests, the SSL certificate state or the response status code are extracted from the ChromeDriver log. The collected data is then transferred to our web server implementation which updates our database accordingly.

### Web server implementation

The Aviator web server was implemented using Python 3.8.5 with Django 2.2.16, Postgres 11.1 and Redis 5.0, all running in fixed environment docker containers. The frontend was styled with Bootstrap 4.5.2. Tables were rendered with DataTables 1.10.19 and plots were visualized with Plotly 1.58.4 and Chart.js 2.9.4. Websites can be either online, temporarily offline, or offline. They are determined to be online only if their response return code is 200. If a web site was online in at least one of the two accesses at one day, the web site was counted as online for that day. The use of analytics or tracking software is determined by querying the driver log file for the following keywords: ‘google-analytics’, ‘matomo’, ‘woopra’, ‘gosquared’, ‘go-squared’, ‘foxmetrics’, ‘fox-metrics’, ‘mixpanel’, ‘heap’, ‘statcounter’, ‘stat-counter’, ‘chartbeat’, ‘clicky’, ‘leadfeeder’ and ‘piwik’. To determine which programming language/framework was used, we inspect—where available—the cookies set by the webpage (PHP, Perl, JavaScript and ASP) as well as the HTML header (Shiny and Dash). The source code is deposited in GitHub and reachable at https://github.com/CCB-SB/Aviator.

### Curated web server APIs

Since the mere availability of a landing page does not guarantee the correct function of a web server, we provide the possibility for web server authors to implement an API endpoint computing the digit sum for the ‘input’ query parameter, provided as a GET request, in their web server. We then query this endpoint in addition to the webpage to monitor the availability of the tool. In addition to the author contact information, tool name and URL, PubMed ID, API endpoint and tool description, authors can provide tags to categorize their tools. We extracted these tags from the EDAM ontology terms (28) used by bio.tools. If they wish, the authors will be automatically contacted after their website is offline for more than a previously selected number of days. We provide API snippets for common programming languages (Perl, PHP, Python, JavaScript) for download. Further, we make an OpenAPI documentation available, as recommended as a best practice (29).

## RESULTS

### Aviator web interface

From the landing page https://www.ccb.uni-saarland.de/aviator users can access all relevant parts, most importantly the general and the curated module. By selecting ‘tool list’, the main general results are listed in a tabular manner and users can select from 16 different features to be shown. Main columns include the title, authors, the journal, abstract, publication year and average as well as current availability. In addition to the original link, the current link can be shown along with many other more detailed features such as RAM usage and whether SSL is included in the web pages. Where available, also a link to bio.tools is displayed. From this list, the main analysis functionality can be initiated. Each feature can be searched and filtered for user-defined values. To keep the web page as clear as possible, the abstract is hidden and only displayed when selected. Nonetheless, the full text search within the abstract is activated. As soon as an analysis is initiated, the statistic plots on the bottom of the page are automatically updated according to the search results. This includes a heatmap showing the availability and a dashboard for the most relevant statistics. Our definition of online, offline and temporarily offline is as follows: if a web site is not accessible for at least 14 consecutive days it is considered as offline (as soon as it is working again it is shifted immediately to the online category). If it is offline and has been reached at least once within the previous two weeks it is considered as temporarily offline, to account for maintenance work and other short-term effects. All other web sites are considered as online. The analyses are performed on a website basis on all websites that are stored in the Aviator database, since one publication can contain more than one website. For each of the entries, a detailed results page can be reached by selecting ‘show details’. This page lists IP address, which analytics platform is embedded (e.g. Google Analytics), the programming language (e.g. PHP), the Security Certificate, frontend and backend loading time as well as RAM consumed by the browser. In addition to tabular results, also graphical representation of the response time (back end and front end) as well as the availability are available. In case of errors, the HTTP error code is displayed. The data can be downloaded as comma-separated values file for further analyses.

On the statistics page we provide many other useful overview plots, such as offline / online and temporarily offline tools split per year, or per journal, availability over weekdays or information on the offline time of tools, i.e. the number of days required for tools to return to service.

### Surveillance tools

The curated module that currently only monitors the tools of the contributing work groups—but is open for registration by any interested user—have the same functionality as the general tool described in the previous section. The only difference is that the availability is not only checked by accessing and downloading the web page. To monitor the tool's functionality more effectively than over their entry page, we provide simple API snippets in different common programming languages that can be integrated into web servers and web-based databases of users. Code snippets are available for download for frameworks in JavaScript, PHP, Perl and Django/Python. The API endpoint computes and returns the digit sum for the ‘input’ parameter provided as a GET request. In case of an error, the user can be notified by email automatically, to minimize down-times. Using these API scripts, we can also monitor whether the backend of a web server is properly working. Again, all analyses and dashboards are also available for this curated module. To add their tool, users can download and integrate the API snippets and then enter the tool via the registration form on the main Aviator page. The request will usually be approved within two working days.

### Covid-19 Use Case

**Figure 1.**
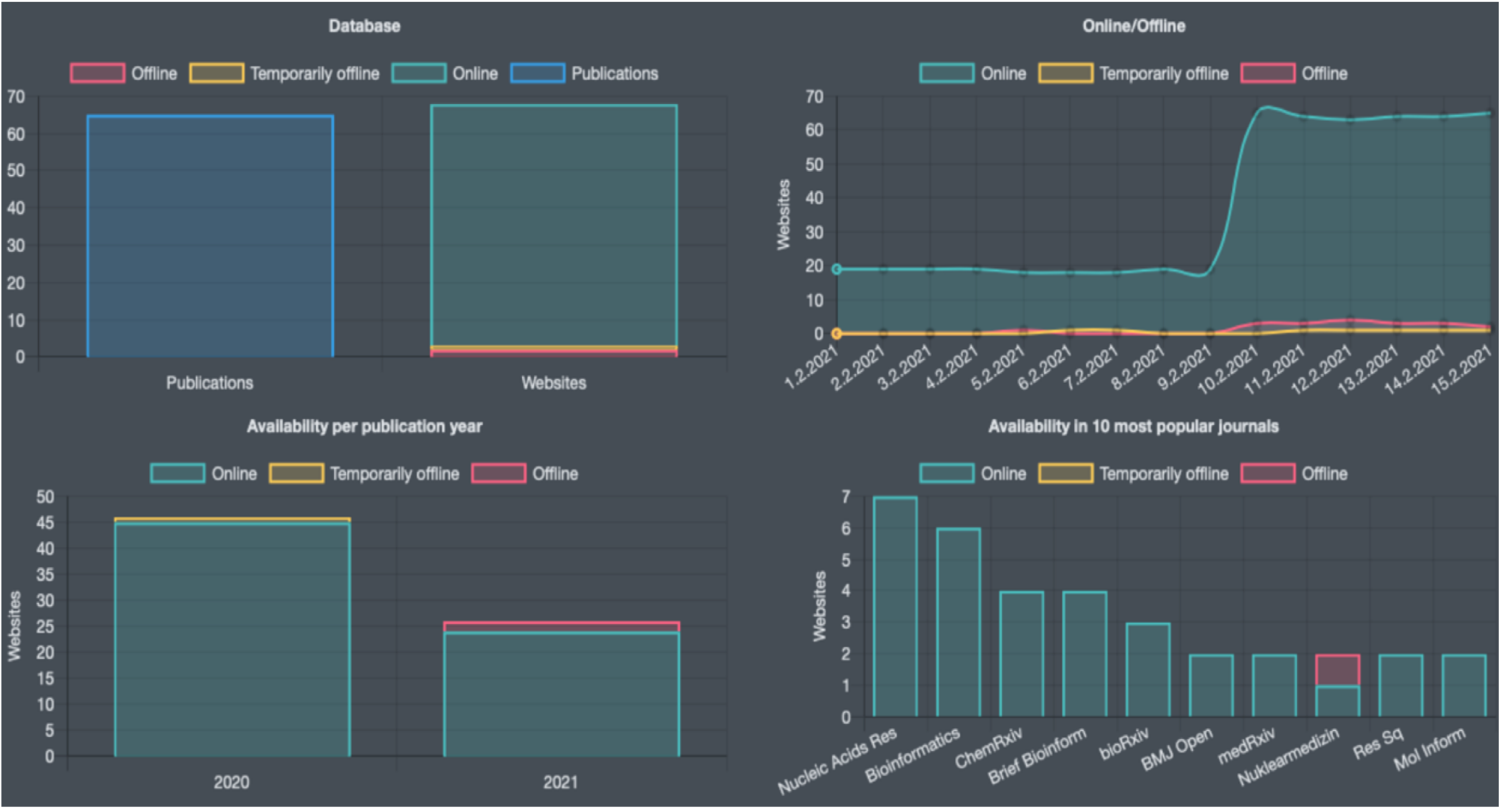
Result of the query for COVID in the general part of Aviatora. Sixty-five publications present links to 72 web sites (upper left part). The largest fraction of those is online. A scheduled update on the data warehouse of Aviator on 10 February reveals a significantly increased number of Covid related databases and tools (upper right panel). As expected, the publications come only from 2020 and 2021 (lower left part). Finally, most of the web sites are published in *Nucleic Acids Research* and *Bioinformatics* (lower right panel).

To demonstrate the function of Aviator we performed a search through the publication abstracts for Covid to display the performance of Covid-19 related web services (Figure 1). The very first release of Aviator (November 2020) only contained 22 web sites, while following an update on February 10^th^ currently 72 web sites are listed. These are matching to 65 different publications. Of the 72 web sites, two are permanently offline, one was temporarily offline and seven others have been offline during the considered period but are now online again (15 February 2021). The remaining 62 websites were always online. The largest fraction of publications (7; 10.7%) has been published in Nucleic Acids Research, followed by Bioinformatics (6; 9.6%) and Briefings in Bioinformatics (4; 6.2%). Also pre-prints from ChemRxiv and bioRxiv are displayed. Altogether, the five most represented sources cover almost 40% of the respective publications. Two websites were developed using the Shiny R package, two with PHP and three with Java. For the remaining websites, the used programming environment could not be detected automatically. Interestingly, 10 weeks later (24 April 2021), the number of permanently offline Covid-19 web servers doubled. Knowing and actively monitoring such trends is of importance for many research aspects.

We selected one of the publications presenting PAGER-CoV (30). This tool uses google-analytics and was permanently online since it has been published. The response times on the frontend site were between 1.8 and 2.4 seconds at a RAM consumption of 15–19 MB. Importantly, the Aviator results page is highly dynamic, Figure 1 shows for example the results obtained on 15 February. Since only the past 30 days are shown in the availability heatmap and the past 14 days in the dashboards, results can vary even from day to day.

### miRNA-target use case

miRNA target interactions are an aspect of research that is frequently applied to infer downstream effects in biomedical studies. Here, a broad range of web services, partially with overlapping functionality, exist (31). If a third-party web service is used in a respective project, the researchers are somewhat dependent from the web service. If for example in the revision phase a web service is not available that whole part of the research study has to be modified. We searched for miRNA target tools, including those that predict single targets, target networks and related tasks. Of 67 tools, 50 were online and 17 were offline, no tool was temporarily offline (data from 24 April 2021). This result seems at first glance not to argue for the utility of Aviator, visiting the web sites would identify the online and the offline tools as well. In fact, however six (12%) of the tools that were online, were offline at least one day within the past month. The most relevant case was a tool that was offline five times with a total duration of eight days. Our previous results suggest that tools that are offline several times have a higher tendency to disappear permanently and researchers might want to avoid using such tools in their studies while selecting an alternative that is more sustainable. Importantly, this application scenario does not only apply to research efforts. In teaching, web services are frequently used as educational tools for students. Here, tools that are permanently available are from our perspective better educational tools.

## DISCUSSION AND FUTURE DIRECTION

With the decreasing entry hurdles to develop web servers and thereby implied expansion of such services an automatic monitoring procedure becomes increasingly important. With Aviator we have implemented such a tool that automatically extracts and monitors web servers from the literature. Our use case demonstrated, that especially for highly dynamic fields, such as COVID-19 research, it is necessary to implement automatic update workflows to keep track of the latest changes. We consider Aviator a long-term project and will extend it over time. For example, we will work on a sophisticated artificial intelligence application to reduce the manual curation effort when finding new web-based tools. This could be integrated in our automatic update routines to reduce false positive hits.

An open issue, which we try to address with the API testing, is to determine if a reachable website is also functional. A step in this direction, to increase the correlation between availability and functionality of a website, could be the integration of an automatic link availability checker. An accumulation of dead links could then potentially be used as hint for a loss of functionality. In the same manner an accumulation of site assets failing to load might indicate malfunction. Additional insight might also be gained through the monitoring of the changes introduced to the web pages and queries over time.

While the automated API testing already improves the determination of the functionality of a web server, it can still be affected by false positives. By monitoring the API test results together with the automatically collected web page accessibility results, we can provide a more precise availability assessment. Further improvements might be made by establishing contact with the web server maintainers to provide access to the tool specific APIs with a set of expected query parameters and corresponding responses. Currently, this feature is used only by a limited number of researchers that all contributed to Aviator. While the results of the automated testing are currently thus by no means statistically significant, we hope to stimulate some interested readers to join the user community to understand whether this automated API testing has an additional value over the more simply website query. Finally, we aim to implement OpenAPI support in the future, which can enable effective functional testing without any need to contact web server owners or to add test-specific code.

We currently collect screenshots of all websites twice a day. An orthogonal approach to the monitoring of the web server accessibility via webpage access, would be to determine if neural networks can identify patterns in those for non-functional web servers or web servers under maintenance. This could enable a more precise availability scoring or even explicit flagging of maintenance frames. In addition, continuous monitoring of multiple variables, such as the access time patterns, might give indications for an imminent maintenance or disappearance of a web server. Within two years, we aim to store website screenshots and data sets of 10,000 websites on average, twice a day and for 730 days, providing us with 17.5 million website screenshots and structured data sets, including the information on whether the websites were permanently online or temporarily offline. Already today, we can build statistics on almost 10 000 websites, 4.5 million website queries and a data collection exceeding 2 TB. We will continue to collect the data and to make use of the data, for example to find patterns which might contribute to a predictive maintenance of web servers. Because of our ambition to have a comprehensive data collection, we are extracting and storing features that have no immediate relevance for the availability. Two examples include the fact whether SSL is implemented and the RAM usage. While there is no obvious connection for these features with the availability, it might be interesting to test the hypothesis whether websites with SSL are less frequent offline than those without SSL. Similarly, one hypothesis could be that websites with higher RAM consumption might be more frequently offline. To make statistically solid conclusions in the future, we collect and display respective features constantly.

In the near-term future, we plan to improve the annotation of the existing data collection. This includes linking the data to the Web of Science, providing additional meta data such as the country or more curated research categories and keywords. Also, citation information for the different tools will be integrated from the Web of Science. Finally, we aim to link Aviator to our scientometric tool Scipe (24,25), facilitating a detailed scientometric analysis of the web repositories. According to our previous research, active surveillance of web services can greatly support their sustainability. With Aviator, we hope to contribute to this topic, to bring the importance of the topic to the attention of researchers and to improve the availability of web servers generally.

## DATA AVAILABILITY

AVIATOR is freely available at https://www.ccb.uni-saarland.de/aviator.
